# Proceedings of Eighth Annual Session of I. H. A.

**Published:** 1888-08

**Authors:** 


PROCEEDINGS OF THE EIGHTH ANNUAL SESSION OF THE INTERNATIONAL HAHNEMANNIAN ASSOCIATION.

The meeting of the International Hahnemannian Association at Long Branch, June, 1887, was an unusually successful one. The papers read there were of great practical value. Hence this neat volume, which the Publication Committee now sends us, is one of interest and value. The delay in its appearance was unfo tunate, but seems to have been due to a combination of circumstances which could not be avoided.

As is usual with the work of this Association, the greater part of its papers are devoted to the practical subjects embraced under the Bureaux of Materia Medica and Clinical Medicine. In this respect the papers of this Association will be found to be in a striking contrast with those of the Institute, or with those of any of the old school medical associations. In both of these classes of medical societies the larger part of the papers will be found devoted to pathology to surgery, and the like, for both of these classes of medical men have little faith in or knowledge of true materia medica or therapeutics. With the members of the I. H. A., their *Homœopathic Materia Medica* is their sole reliance for the treatment of all diseases, and hence the time and study given to it.

Many of these best papers, with the discussions upon them, were given in this journal last year, therefore we need do no more now than merely mention a few of the most important contents of this volume.

Beginning with the Bureau of Materia Medica, we find a paper by the late Dr. Ad. Lippe, entitled, "Progressive Materia Medica: How it is Developed."

The next paper is one by the veteran P. P. Wells, upon "Errors In Drug Proving," a most important subject; some of our would-be provers would do well to read this paper. A paper by Dr. Harlyn Hitchcock, on "Homœopathy and its Relation to the Germ Theory," deserves careful study. Dr. E. B. Nash gives one of his practical papers upon "Tissue Remedies," which, with the discussion thereupon, we will give in full in our next issue. It will be seen that these "tissue remedies" are to be prescribed as are all drugs, only when indicated by their symptoms. Further papers were presented by Dr. W. P. Defriez on clinical "Confirmation;" by Dr. Biegler on "Verifications"—both useful papers. Dr. H. C. Allen gives two excellent provings, even though partial—one on Salicylic acid, the other on Melilotus alba. Dr. Allen is to be especially commended for his work in this line. Dr. McNeil also gives some of his experiences under the title of "Confirmations." A further proving of "Aqua Sanicula," with some clinical cases, is given by Dr. Sherbino. In this issue will be found other clinical confirmations of this drug by the same observer. Papers upon Dulcamara are presented by Drs. McNeil, Clark, Wells, and Wesselhœft. Besides a paper upon this drug, Dr. Wesselhœft presents some provings of it made upon female provers.

The excellent repertory on gonorrhœa, etc., by Dr. S. A. Kimball, belongs to the work of this bureau, but as it has been published separately it is not given in this volume. Under the report of the Surgical Bureau, we find the famous paper of Dr. Julia Morton Plummer upon "Antisepticism," which was received with such favor. The "History of a Mammoth Ovarian Tumor Successfully Removed" is presented by Dr. E. Carleton—only a thorough homœopath could do such work as shown in that case. Drs. Sherbino, Lowe, and Stowe have papers under this bureau, all of them good ones, showing what homœopathic medication can do in these surgical cases.

Under the Bureau of Clinical Medicine a great variety is given. Most of these papers have been published in our last year's volume. A lengthy paper on Variola, with a repertory, is given by Dr. W. J. Guernsey. Dr. C. W. Butler gives one f his interesting papers, this time upon "Sulphuric acid."

We should like to be able to give a *resume* of some of these papers, for they are well deserving of careful study by all who desire to learn more of the homœopathic materia medica and its therapeutics. This volume is perhaps the best the Association has yet issued. It is very neatly printed, and is altogether highly creditable to the Association. We believe the thanks of the members are due to Dr. H. Hitchcock, who superintended the publication of the volume.

